# Aggressiveness pattern and second primary tumor risk associated with basaloid squamous cell carcinoma of the larynx

**DOI:** 10.18632/oncotarget.21327

**Published:** 2017-09-28

**Authors:** Filippo Ricciardiello, Michele Caraglia, Brigida Iorio, Teresa Abate, Mariarosaria Boccellino, Giuseppe Colella, Flavia Oliva, Pierpaolo Ferrise, Silvia Zappavigna, Mario Faenza, Giuseppe A. Ferraro, Giulio Sequino, Giovanni Francesco Nicoletti, Massimo Mesolella

**Affiliations:** ^1^ Division of Otolaryngology, “A. Cardarelli” Hospital, Naples, Italy; ^2^ Department of Neurological, Reproductive and Odontostomatological Sciences, University “Federico II” of Naples, Naples, Italy; ^3^ Department of Biochemistry, Biophysics and General Pathology, University of Campania “L. Vanvitelli”, Naples, Italy; ^4^ Department of Medical, Surgical and Dental Specialties, University of Campania “L. Vanvitelli”, Naples, Italy

**Keywords:** laryngea lbasaloid squamocellular carcinoma (BSCC), prognosis of laryngeal basaloid squamous cell carcinoma, mortality of laryngeal BSCC, loco-regional recurrence in laryngeal BSCC

## Abstract

Basaloid squamous cell carcinoma (BSCC) is a rare, aggressive and distinct variant of squamous cell carcinoma (SCC) of the upper respiratory and digestive tract. We have evaluated disease specific survival (DSS) and overall survival (OS) through Kaplan-Meier method and mortality risk through univariate statistical analysis of Cox in 42 cases of BSCC and other 42 of laryngeal SCC (LSCC) matched for both age and sex. We demonstrated that laryngeal BSCC is a more aggressive tumor than LSCC as is associated to higher nodal recurrence of pathology (*5 vs 2* patients, overall risk, OR 2.7), a reduced survival (median survival 34 vs 40 months, OR 3.2 for mortality); in addition, basaloid patients have a higher risk to be affected by second primary tumors (13 vs 3 patients, OR 5.8) and a higher probability to die for this second tumor (Hazard Risk, HR 4.4). The analysis of survival shows an increased mortality risk concurrent with the parameters assessed by univariate analyses that assume a predictive and statistical significance in second tumor and grading in basaloid LSSC.

## INTRODUCTION

Basaloid squamous cell carcinoma (BSCC) is a high-grade variant of squamous cell carcinoma (SCC) of the upper respiratory and digestive tract [1–4]. It was firstly described in 1986 by Wain et al. as a distinct histological variant of SCC and recognized by the World Health Organization (WHO) as a peculiar entity in 1991 [9]. This malignancy seems to have a predilection for the head and neck region, especially for supraglottic larynx, tongue and hypopharynx [2, 3], but it can also occur at other sites such as esophagus, lung, thymus, anus, penis and cervix [3, 4, 7]. SCC basaloid subtype represents less than 1% of laryngeal carcinomas; it affects mainly men in sixth or seventh decade of life, and differs from the common form of SCC with specific morphological and biological features [4, 5]. BSCC has a poor prognosis for its invasive growth pattern and for its frequent diagnosis in advanced stage characterized by cervical node metastases [4–7]. Metastases in loco-regional nodes are reported in 64% of patients and visceral metastases (lung, liver, bone, brain and skin) in 44% of cases [10]. The diagnosis is often delayed so that reasonable treatment options are radical surgery, chemoradiation or radiation alone (5 years survival is reported to be 17.5%) [11, 12]. In this study, we have analyzed the aggressiveness of laryngeal BSCC in a group of 42 patients (basaloid subgroup A) compared with a homogeneous group of 42 patients affected by laryngeal SCC (non-basaloid subgroup B) evaluating survival through Kaplan-Meier method, mortality risk through univariate statistical analysis of Cox considering the following variables: staging, grading, second tumors and treatment strategy. Moreover, we have studied any significant variable influencing survival through a multivariate and Hazard Risk (HR) analysis. In addition, we have calculated the overall risk (OR) for second tumors, mortality, nodal recurrence of pathology (N Recurrence) and recurrence of tumor (T Recurrence).

## RESULTS

### Demographic and clinical characteristics of the patients

Study population was formed by 84 cases of laryngeal carcinoma (74M and 10F) divided into two subgroups: 42 cases of basaloid laryngeal squamous cellular carcinoma (LSCC) (A) and 42 cases of Non-basaloid LSCC (B). Basaloid subgroup (A) was composed by 37 males and 5 females with a median age of 71 years (range: 37-86 years); non-basaloid subgroup (B) by 37 males and 5 females with a median age of 71 years (range: 40-82 years). No differences were recorded in staging distribution between the two subgroups; in fact, in both subgroups 2 patients were stage I, 3 stage II, 20 stage III and 17 stage IV according to VII edition of TNM [13], respectively. Also for histological grading data there were no differences between the two subgroups; in fact, no patient had a well differentiated carcinoma (G1), 6 patients had a moderately differentiated (G2), 10 patients a G2-G3 and 26 a poorly differentiated carcinoma (G3), respectively. Treatment strategies were the following: 5 patients underwent to cordectomy, 8 to a supraglottic laryngectomy, 5 to subtotal laryngectomy and 24 to a total laryngectomy, respectively. In all cases of open surgery we performed also a neck dissection. Follow-up was performed in all patients; in basaloid subgroup median follow-up was 38 months (95% CI: 30.72-48.00 months), in non-basaloid subgroup 42.50 months (95% CI: 32.72-49.27 months). The analysis of follow-up did not show any statistically significant difference between these two subgroups (p= 0.77), using Wilcoxon/Mann-Whitney test for independent and non-parametric variables. Therefore, basaloid and non-basaloid subgroups were homogeneus for demographic features, staging, grading, treatment strategy and follow-up.

### Clinical outcome of the patients

#### Survival analysis

**Figure 1:**
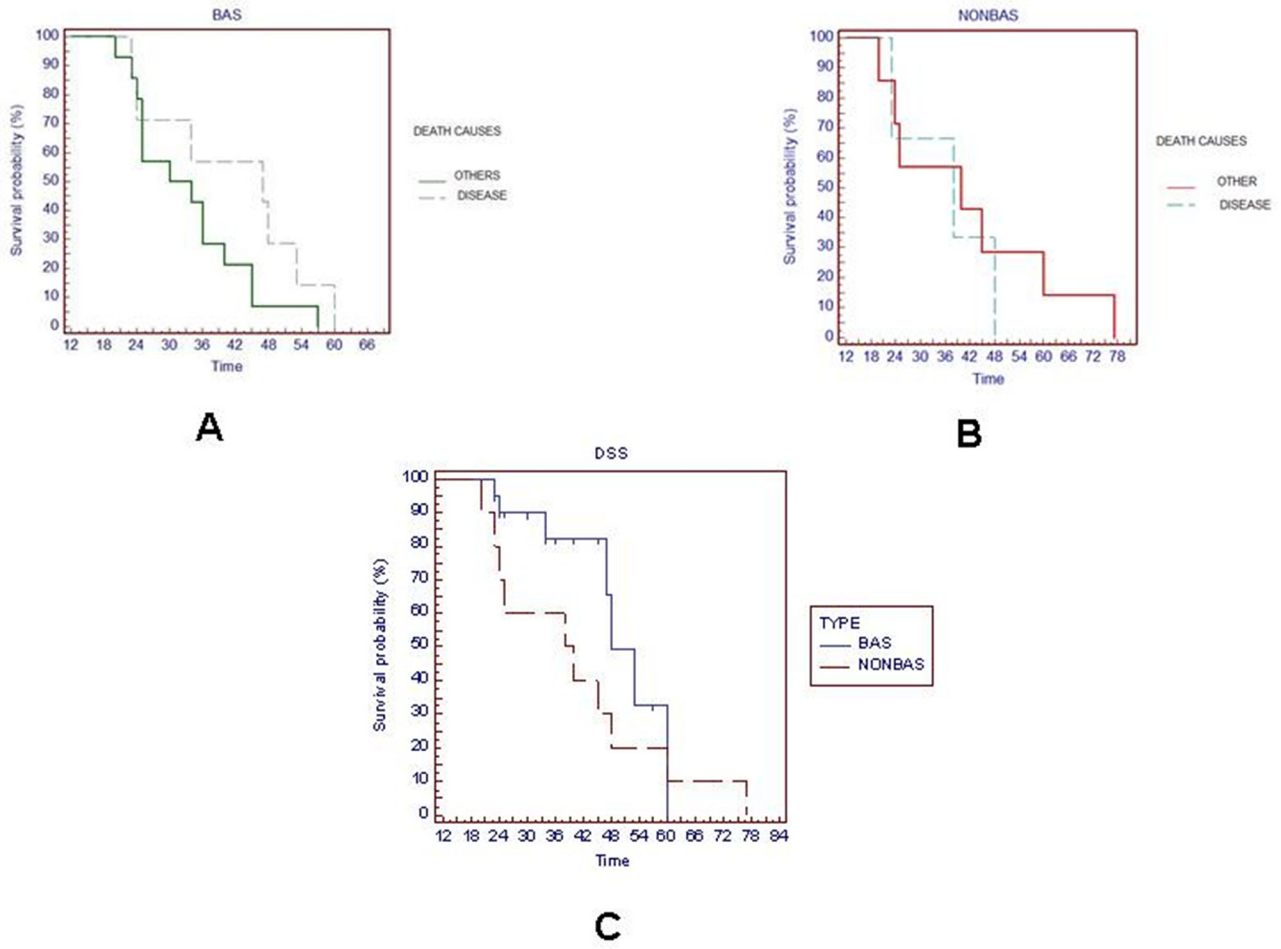
Survival rate analysis in basaloid subgroup A **(A);** in non-basaloid subgroup B **(B);** disease specific survival comparison between two subgroups **(C).**

**Figure 2:**
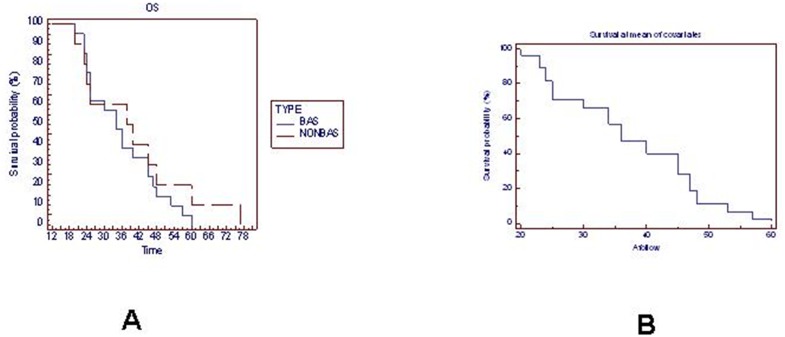
OS in basaloid A and non-basaloid B subgroups **(A);** survival probability in relation to follow-up period in months in basaloid subgroup B **(B).**

In basaloid subgroup, 14 patients died for other diseases (including second tumours) and 7 for cancer originating in laryngeal site; the median survival was 34 months in the first group of patients (deceasing for other diseases) and 47 months for patients deceased for cancer originating in laryngeal site, respectively. Analysis of the survival rate in this subgroup of patients (basaloid subgroup A) analyzed for death causes (other diseases vs cancer originating in laryngeal site) showed a not statistically significant *p value* (2.1575 Chi-square, p= 0.1419; HR 1.8234; 95% CI 0.7872 to 5.3145) as shown in Figure 1A. In non-basaloid subgroup B, 7 patients died for other diseases (including second tumours) and 3 for cancer originating in laryngeal site, respectively. Also in this subgroup the difference in the survival rate for death causes was not significant (0.3158 Chi-square, p= 0.5742; HR 0.6914, 95% CI 0.1375 to 3.0038). In fact, median survival was 40 months for the first ones and 38 months for patients deceased for cancer originating in laryngeal site, respectively (Figure 1B). Comparing the specific disease survival of the patients deceased for laryngeal cancer between basaloid (subgroup A) and non basaloid subgroups (subgroup B) we did not find again any statistically significant difference (2.1515 Chi-square, *p*= 0.1424; HR 0.5317 95% CI 0.1513 to 1.3123). Median survival was 48 months for subgroup A and 40 months for subgroup B, respectively. As second tumors were not considered in disease specific survival, this explains this apparent discordance (Figure 1C). Moreover, the overall survival between basaloid and not basaloid subgroup was not significantly statistically different. Median survival was 34 months for basaloid subgroup and 40 in non-basaloid (0.8092 Chi-square, p= 0.3683; HR 1.3634 95% CI 0.6486 to 3.2140), respectively (Figure 2A). Figure 2B shows survival probability in relation to follow-up period in months in basaloid subgroup B. Cox proportional hazard model was used to verify if staging, grading, disease specific survival, treatment strategy and second tumors were independent prognostic factors both for basaloid LSCC patients (subgroup A) and for non-basaloid LSCC patients (subgroup B).

Univariate analysis showed that second tumor occurrence (HR 4.3634, p= 0.01583 for subgroup A and HR 3.9333, p= 0.07462 for subgroup B, respectively) and high G3 grading (HR 3.9333, p= 0.03 for subgroup A and HR 1.5246, p= 0.5533 for subgroup B, respectively), but not staging (HR 0.85, p= 0.62 for subgroup A and HR 0.7911, p= 0.6302 for subgroup B, respectively), disease specific survival (HR 0.49, p= 0.15 for subgroup A and HR 1.507, p= 0.5768 for subgroup B, respectively) and treatment strategy (HR 0.8494, p= 0.45 for subgroup A and HR 0.6521, p= 0.1306 for subgroup B, respectively) correlated to decreased survival only in patients with basaloid LSCC (Table 1). Multivariate analysis of the same variables showed that again only second tumour occurrence had a statistically significant trend in correlating to poor prognosis score (p=0.07) while the grading was not statistically significant (p=0.29, HR for second tumor 3.18 and for grading HR 2.44, respectively) (Table 2). It was not possible to perform multiparametric analysis in LSCC because no variable was significant in univariate analysis.

**Table 1:** Demographic, clinical and pathological characteristics of enrolled patients

Variable	Basaloid subgroup-A	Non-bas subgroup B
**Median Age**	71 y (37-86)	71 y (40-82)
**Sex**		
Male	37	37
Female	5	5
**Stage**		
I	2	2
II	3	3
III	20	20
IV	17	17
**Grading**		
G2	6	6
G2-G3	10	10
G3	26	26
**Treatment strategy**		
Cordectomy	4	5
Supraglottic laryngectomy	8	8
Subtotal laryngectomy	4	5
Total laryngectomy	26	24
**Second tumor occurrence**	13	3
Lung	6	2
Colon	7	1
**Death**	21	10
**Cause of death**	21 patients	10 patients
Laryngeal cancer	7	3
Second tumor	12	3
Other causes	2	4
**Locoregional recurrence**	6	3
T recurrence	1	1
N recurrence	5	2
**Median Follow-up (months)**	38 (95% CI 30.72-48)	42,50 (95% CI 32.72-49,27)
**Total patients**	42	42

**Table 2:** Univariate analysis of clinical and pathological characteristics between the subgroups

Variable	Basaloid subgroup A	Non-basaloid subgroup B
**Second tumor occurrence**		
p value	0.008	0.62
HR	4.3634	1.4306
95% CI of HR	1.3265 to 14.3525	0.3459 to 5.9167
**DSS**		
p value	0.15	0.5768
HR	0.49	1.507
95% CI of HR	0.1807 to 1.3288	0.3596 to 6.3151
**Grading**		
p value	0.03	0.5533
HR	3.9333	1.5246
95% CI of HR	0.8795 to 17.5909	0.3808 to 6.1034
**Staging**		
p value	0.62	0.6302
HR	0.85	0.7911
95% CI of HR	0.4473 to 1.6152	0.3062 to 2.0440
**Treatment strategy**		
p value	0.45	0.1306
HR	0.8494	0.6521
95% CI of HR	0.5638 to1.2795	0.3757 to 1.1319

Legend: HR, Hazard Ratio; CI, Confidential Interval; DSS, Disease-Specific Survival

## DISCUSSION

BSCC was firstly described in 1986 by Wain et al., who reported 10 cases localized in the tongue, larynx and hypopharynx as a distinctive histological variant of SCC with aggressive behavior [9]. BSCC has been defined in the “2005 WHO blue book” as an aggressive high grade variant of SCC composed of both basaloid and squamous components [14] and it is believed to arise from a totipotent primitive cell in the basal layer of the surface epithelium or from the salivary duct lining epithelium [1, 15]. Upper aerodigestive tract is the most common site of origin of this tumor in the head and neck [15], especially larynx (supraglottic larynx in 68.6% of cases, epiglottis) [16], hypopharynx (piriform sinus), base of the tongue and less frequently the oropharynx (palatin tonsils) [1, 8, 15, 17]. A recent review of the literature shows that BSCC appears in both sexes but predominates in males (14:1) between 60 and 80 years old [15]. The tumor shows the same strong association with tobacco and alcohol as conventional SCC [8, 15, 18]. It is still unclear the correlation with Epstein-Barr virus (EBV) and human papilloma virus (HPV) as a causal or contributory factor in BSCC [18]. Clinical signs and symptoms are not specific and related to tumor location [14]. The basaloid subtype of SCC is less than 1% of laryngeal carcinoma [1, 4]. The tumor can also occur at other sites such as the esophagus, lung, thymus, anus and cervix. Pathologically, BSCC has a distinctive biphasic histologic appearance, including basaloid and *in situ* or invasive SCC components [1, 19]. Some histological features such as nuclear pleomorphism, hyperchromasia, mitotic activity, necrosis and perineural invasion, when occurring all together indicate a high grade malignancy [7, 19]. By immunohistochemistry, BSCC expresses cytokeratins (AK1, AK3, LP34) and epithelial membrane antigen EMA [15]. Some authors recommend cocktails of keratins composed of CAM 5.2, pankeratin AE/AE3 and CK7, while others propose the high molecular weight keratin 34βE12 as the most useful marker for this tumor. There is no specific immunohistochemical pattern to distinguish this tumor from others. However, quite recently Coletta et al. have demonstrated the importance of cytokeratins 1, 7 and 14 in the diagnosis of BSCC and Emanuel et al. have stressed the value of p63 in making a distinction between BSCC and adenoid cystic carcinoma of the head and neck, showing that p63 positivity is diffuse in BSCC and partial in adenoid cystic carcinoma [15, 20, 21]. The most important differential diagnosis for BSCC is the solid variant of adenoid cystic carcinoma and small cell neuroendocrine carcinoma [4]. Adenoid cystic carcinoma is positive for smooth muscle actin, whereas BSCC is negative; the latter has also greater reactivity for vimentin compared to BSCC [7]. Synaptophysin and chromogranin are positive in small cell neuroendocrine carcinoma, but are consistently negative in BSCC [4]. Kleist et al. and El-Mofty et al. have detected a higher frequency of HPV and HSV in basaloid tumors than in conventional SCC of head and neck especially in some locations like nasopharynx, but other authors have not found any difference especially in larynx, such as in our experience about laryngeal cancer [15, 22, 23, 24]. The supposed higher clinical aggressiveness of BSCC compared to the conventional SCC is still controversial. Winzenburg et al. were the first to correlate some histological variables of BSCC to prognosis, reporting that cases with pure basaloid histology and comedonecrosis showed distant metastases in 46% of the patients. A predominantly basaloid histology was associated with distant metastases in 52% of their patients [24, 25]. Patients usually present at an advanced stage: in fact, some authors reported 76% in Stages III or IV at diagnosis, while others described that 36% of cases were at Stage III and 41% at Stage IV. Thankappan reported 45.9% of nodal metastasis at presentation and no distant metastases [16, 26]. Generally, distant metastases occur also in 10-75% of BSCCs and lung is the main organ for distant metastases in BSCC [27]. Since most cases of BSCC occurred in advanced stage, this feature is believed to be reflective of tumor aggressiveness and poor prognosis [1]. However, with the introduction of new treatment modalities for head and neck tumors, the disease-free time is increased, resulting in a higher detection of distant metastases. The incidence of distant metastases in patients with laryngeal cancers remains low, with a rate of 5% reported by Bahar et al. [18]. On the other hand, finding a second primary tumor is not uncommon in head and neck BSCC. McKay and Bilous in 1989 reported a case of BSCC of the hypopharynx with microinvasive squamous cell carcinoma in the arytenoid region [28]. In 1991 Seidman et al. presented a series of 11 BSCCs, two of which (tumors arising in the piriform sinus and vallecula) with synchronous SCC in upper gastrointestinal tract (oesophagus and palate, respectively) [28, 29]. According to Ereno et al., 2008, the incidence of metachronic second primary tumors was 15%, 6/40 cases, in details 3 in lung, 3 in bone and 1 in liver, respectively [15].

In the present study, we have observed second primary tumors in 13 out of 42 patients (30.9%) in patients with laryngeal BSCC and in 3/42 (7.1%) patients with LSCC, particularly in lung and colon. Mortality was 21/42 in BSCC (50%) and 10/42 in LSCC (23.81%):

∙ 7/21 patients of BSCC (33.33%) died for laryngeal cancer;

∙ 12/21 (57.14%) of BSCC died for second tumor;

∙ 2/21 (9.53%) patients of BSCC died for other causes.

∙ 3/10 patients of LSCC (30%) died for laryngeal cancer;

∙ 3/10 patients of LSCC (30%) died for second tumor;

∙ 4/10 patients of LSCC (40%) died for other causes.

Conversely, observed survival was 50% in BSCC and 76,19% in LSCC.

According to our results these data were statistically relevant; moreover, BSCC had a 4.4-fold higher risk to be affected and dying for a second primary tumor than the patients with LSCC (HR 4.4); when these data were statistically corrected for grading the HR reduced to 3.2-fold, likely because patients with higher grade tumor decease before developing a secondary tumor. Regarding the grading, patients with laryngeal BSCC had a risk to die for laryngeal cancer 3.9-fold higher than patient with LSCC; when these data were statistically corrected for second tumor the HR reduced to 2.4-fold, probably because some patients with high grade laryngeal BSCC die for a second tumor. Regarding treatment, in literature some authors perform surgery alone in 28.5% of patients with BSCC of larynx; combined modality treatment is administered in 67% of cases [27]. Total laryngectomy is the most common surgical treatment [16]. BSCC has a poor prognosis and its invasive growth pattern as small solid foci may be misdiagnosed in small biopsies [1]. These patterns justify, in our view, radical treatment measures. Finally, the currently practiced treatment strategies apparently have no effects on the outcome, and more specific anti-basaloid chemotherapy medications should be developed. There are not statistically significant differences between survival in basaloid and non-basaloid subgroups for the cancer originating from larynx but in basaloid group there was a high death risk for second tumors. A high grading was also associated with mortality risk.

## MATERIALS AND METHODS

### Patients

Study population was selected among patients of Ear-nose-throat (ENT) Units at Federico II University and Cardarelli Hospital, Naples (Italy) from 1^st^ January 2006 to 31^st^ October 2015. Patients enrolled in the study underwent to ENT examination by laryngoscopy, neck and chest CT with intravenous contrast, biopsy with histological examination that confirmed diagnosis of laryngeal cancer. Patients were treated with cordectomy performed with CO_2_ laser aid or open laryngectomy (supraglottic, subtotal or total). In post-operative period, patients underwent to oncological follow-up, examinations by ENT specialist by direct fiber optic laryngoscopy and video recording in accordance with the timetable guidelines for each tumor stage, annual neck and chest CT and total body PET for the control of both loco-regional and distance disease recurrence. pTNM analysis was made according to the criteria of VII edition of TNM [12]. Patients with follow-up lower than 12 months were excluded from the study. According to these inclusion criteria, the study population was formed by 84 cases of laryngeal carcinoma (74M and 10F) divided into two subgroups: 42 cases of basaloid LSCC (A), according to the histological criteria proposed by Wain et al. [9], and 42 cases of non-basaloid LSCC (B). Population of basaloid subgroup (A) was composed by all consecutive cases (42) with definitive diagnosis of basaloid carcinoma of larynx (diagnosis relatively less frequent) from 1^st^ January 2006 to 31^st^ October 2015. In order to highlight the features of BSCC, this subgroup was compared to the same number of cases of non-basaloid squamous infiltrating laryngeal carcinoma (B) with negative anamnesis for any other malignant tumors and for exposure to environmental risk factors in work places. Basaloid and not Basaloid subgroups were homogeneous in staging, grading and treatment strategy. The demographic characteristics and staging, grading and treatment strategies for each subgroup of patients are described in Table 3. The study was performed in accordance to the guidelines of the Institutional Ethics Committee, Italian law, and the Declaration of Helsinki, as required for studies based on retrospective analyses on routine archival formalin-fixed, paraffin embedded tissue. All patients provided a written informed consent regarding the use of these data for research puproses.

**Table 3:** Multiparametric analysis in basaloid subgroup

Value	Second tumour occurrence	Grading
p value	0.0725	0.2915
HR	3.1886	2.4445
95% CI of HR	0.9053 to 11.2306	0.4684 to12.7578

### Statistical analysis

Follow-up period of both subgroups was compared through Wilcoxon/Mann-Whitney test for independent and non-parametric variables using Med-Calc software, version 9.3.7.0. We applied the Kaplan-Meier method, normalizing the different categories by the long-rank Mantel-Haenszel test, in order to study overall and disease specific survival (OS and DSS) in each subgroup and to compare them in the two subgroups. Moreover, we calculated the additional risk of second tumor occurrence, T and N recurrence and mortality in patients with basaloid LSCC compared with non-basaloid LSCC by Odds Ratio (OR). A Cox proportional hazard model was used to study the simultaneous contribution of multiple factors to mortality risk. The assessed variables were staging, grading, treatment strategies and disease specific survival in each subgroup. We performed Multivariate Cox regression analysis for significant variables found in univariate analysis (second tumor, and grading) to underline HR of patients with basaloid LSCC. In each test, the p value <0.05 was considered statistically significant.

## CONCLUSIONS

BSCC is a tumor characterized by a biphasic pattern of growth and has an aggressive clinical behaviour. Immunohistochemistry may be helpful in diagnosis. Second primary tumors are frequent. In this study, we have analyzed pattern of aggressiveness of BSCC of larynx compared to LSCC. Second tumor and grading correlated to increased mortality risk in univariate analyses only in basaloid LSSC (group A). Second tumour occurrence together with grading were the only independent prognostic factors for patients with basaloid LSCCs, and were significantly associated with poor prognosis. This correlation was not found, on the other hand, in non basaloid tumours (subgroup B). Presently, the molecular bases of the different biological and clinical behaviors of the two different histological subtypes are not known and this deserves additional investigations. The different expression of molecules involved in the regulation of cancer cell growth and mestatization (i.e.: p63 and cytokeratins) suggests a different molecular feature of this tumour.

Finally, due to both BSCC poor prognosis and higher risk of second tumour occurrence, a strict follow-up with advanced imaging techniques (i.e.: total body PET-TC) is mandatory and strongly recommended for the correct management of these patients.
